# Development of a model for predicting the 4-year risk of symptomatic knee osteoarthritis in China: a longitudinal cohort study

**DOI:** 10.1186/s13075-021-02447-5

**Published:** 2021-02-26

**Authors:** Limin Wang, Han Lu, Hongbo Chen, Shida Jin, Mengqi Wang, Shaomei Shang

**Affiliations:** 1 School of Nursing, Peking University, 38 Xueyuan Road, Haidian District, Beijing, 100191 China; 2 School of Public Health, Peking University, 38 Xueyuan Road, Haidian District, Beijing, 100191 China

**Keywords:** Knee osteoarthritis, Risk, Prediction model

## Abstract

**Objectives:**

We aimed to develop a model for predicting the 4-year risk of knee osteoarthritis (KOA) based on survey data obtained via a random, nationwide sample of Chinese individuals.

**Methods:**

Data was analyzed from 8193 middle-aged and older adults included in the China Health and Retirement Longitudinal Study (CHARLS). The incident of symptomatic KOA was defined as participants who were free of symptomatic KOA at baseline (CHARLS2011) and diagnosed with symptomatic KOA at the 4-year follow-up (CHARLS2015). The effects of potential predictors on the incident of KOA were estimated using logistic regression models and the final model was internally validated using the bootstrapping technique. Model performance was assessed based on discrimination—area under the receiver operating characteristic curve (AUC)—and calibration.

**Results:**

A total of 815 incidents of KOA were identified at the 4-year follow-up, resulting in a cumulative incidence of approximately 9.95%. The final multivariable model included age, sex, waist circumference, residential area, difficulty with activities of daily living (ADLs)/instrumental activities of daily living (IADLs), history of hip fracture, depressive symptoms, number of chronic comorbidities, self-rated health status, and level of moderate physical activity (MPA). The risk model showed good discrimination with AUC = 0.719 (95% confidence interval [CI] 0.700–0.737) and optimism-corrected AUC = 0.712 after bootstrap validation. A satisfactory agreement was observed between the observed and predicted probability of incident symptomatic KOA. And a simple clinical score model was developed for quantifying the risk of KOA.

**Conclusion:**

Our prediction model may aid the early identification of individuals at the greatest risk of developing KOA within 4 years.

## Background

Knee osteoarthritis (KOA) is among the most common chronic diseases leading to disability worldwide, carrying a substantial and increasing health burden [1, 2]. The prevalence of symptomatic KOA and radiographic KOA in patients over 60 years of age ranges from 10.0 to 16.0% and 35.0 to 50.0% [3–7], respectively. Approximately 250 million people have KOA worldwide, with a twofold increased prevalence in men and a threefold increased prevalence in women in the USA over the past 20 years [5]; symptomatic KOA affects approximately 15.1 million individuals in the US population [8]. The estimated number of individuals over 60 years old suffering from symptomatic KOA reached 37.35 million in China [9]. The years lived with disability (YLDs) caused by osteoarthritis ranked tenth in China in 2016 [10] and fourth in South Korea in 2015 [11]. Osteoarthritis had the fifth greatest relative increase in total YLDs from data of six Nordic countries from 1990 to 2015 [12]. KOA was the first leading among osteoarthritis, accounting for 87% YLDs of osteoarthritis [13]. The increasing prevalence of KOA has increased the socioeconomic burden for affected individuals and healthcare systems [14].

To date, there are no effective therapeutic strategies for KOA. Prediction models for KOA aim to synthesize multiple factors to comprehensively predict the incident risk and may allow for early detection and prevention [15]. The Nottingham KOA model was an early model for the prediction of 12-year KOA risk in middle-aged adults, including easily obtainable factors such as age, sex, family history, body mass index (BMI), occupational risk, and history of knee injury [16]; however, it was developed using data from only two communities in the UK, rather than a random sample of the general population, limiting its validity in other populations [17]. Several studies have developed prediction models based on genomic data [18–21] or radiographic/clinical biomarkers [22] such as hip α-angle and spinal bone mineral density. However, use of these models is limited due to their high cost or complexity [23].

Primary risk factors for incident KOA include advanced age, female gender, overweight/obesity, knee injury, and smoking [24–27]. Smoking decreases the risk of KOA, while the other factors increase the risk. Although physical activity [28, 29], occupational factors [24], ethnicity, and genetics [25] have also been associated with the incidence and/or progression of KOA, previous studies have reported inconsistent results due to methodological differences. Other potential risk factors for the development of KOA include metabolic syndrome [30–32], waist circumference [33], and depressive symptoms [24, 34], although findings regarding these factors remain controversial. Previous studies have reported a dual association between osteoarthritis and certain comorbidities (e.g., hypertension, ischemic heart disease, diabetes) [24, 25], suggesting that these comorbidities can influence the incidence and progression of KOA. Existing risk models of KOA have failed to include these potential risk factors, and there are currently no models for predicting KOA risk in the Chinese population. In this study, we aimed to develop a model for predicting the 4-year risk of KOA based on survey data obtained via a random, nationwide sample of Chinese individuals. This model would consider the potential risk factors.

## Methods

### Study design and data source

The present retrospective cohort study relied on 4-year data from the China Health and Retirement Longitudinal Study (CHARLS)—a nationwide study among Chinese adults aged 45 years or older for whom the detailed cohort profile has been published [35]. The national baseline survey for the study was conducted between June 2011 and March 2012 (CHARLS2011), and 17,708 respondents across 150 counties/districts and 450 villages/resident committees were recruited using a multistage sampling strategy. The respondents are followed up every 2 years via face-to-face computer-assisted personal interviews. Detailed information related to demographic background, socioeconomic status, biomedical findings, health status, and functioning was collected at baseline and at each follow-up using a structured questionnaire [35]. Blood samples were also obtained at each time point. The present study included participants recruited in CHARLS2011 and re-examined in CHARLS2015.

### Participants

In this study, the unit of analysis was the person. Individuals who did not suffer from symptomatic KOA in CHARLS2011 and had complete diagnosis of symptomatic in CHARLS2015 were included. Participants who had no complete diagnosis of symptomatic KOA in CHARLS2011 or in CHARLS2015 were excluded. We also excluded those who had over 50% of predictive variables unavailable.

### Outcomes

The primary outcome was the incident of symptomatic KOA during the 4-year follow-up period, and the subject was the unit of analysis. In accordance with the definition utilized in a previous study [36], symptomatic KOA was defined as both physician-diagnosed arthritis and the presence of concurrent pain in either knee joint. The incident of symptomatic KOA was defined as the participant being free of symptomatic KOA in CHARLS2011 and diagnosed with symptomatic KOA in CHARLS2015. The presence of pain in the knee joint was assessed based on responses to the following question: “Are you often troubled by pain in any part of your body?” If the participant answered in the affirmative, the following question was asked: “In what part of your body do you feel pain?”

### Predictor variables

In CHARLS2011, data related to demographic background, socioeconomic status, biomedical findings, and levels of blood biomarkers was extracted. We included the following risk factors highlighted in previous studies and imputed missing values when necessary. The demographic variables included gender, age (year), BMI (categorized as underweight [< 18.5 kg/m^2^], normal [18.5–24.9 kg/m^2^], overweight [25.0–29.9 kg/m^2^], obese [≥30.0 kg/m^2^]), waist circumference (cm), and residential area (urban vs. rural). Waist circumference was categorized into four groups: < 85/80 cm, < 90/85 cm, < 95/90 cm, and ≥ 95/90 cm in men/women. The first group was referred to as the normal group and other three groups were referred to as central obesity based on the diagnosed criteria of central obesity recommended by the Department of Disease Control at the Ministry of Health [37]. The behavior variable included smoking status and engagement in vigorous/moderate/light physical activity. The physical activity score was calculated by multiplying the code for the duration by the code for frequency during 1 week [38]. According to physical activity score, the physical activity was divided into three levels (none, 0; low, 1–4; moderate-to-high, ≥ 5). Health-related variables included history of hip fracture, number of other diagnosed comorbidities, metabolic syndrome (MS) in accordance with Chinese Diabetes Society (CDS) criteria [39], depressive symptoms based on Center for Epidemiologic Studies Depression Scale (CESD-10) score [40], self-rated health status and self-reported difficulties with activities of daily living (ADLs) [41], or instrumental activities of daily living (IADLs) [42]. The list of potential predictors is presented in Supplementary Box 1, along with detailed information related to how each predictor was assessed and the used tools.

### Statistical analysis

#### Model structure

In CHARLS2011, physical activity measures were randomly available for 3684 participants, while blood samples were available for 11,847 participants. Hence, physical activity scores of vigorous/moderate/light physical activity and MS were the main predictors with missing values. The percentage of missing values across the predictors varied between 0.04 and 57% in this study. We assumed data were missing at random and imputed 50 datasets based on the multiple imputation by chained equations (MICE) procedure [43]. The MICE technique improved the data accuracy, as any reasons for missing data could be explained by the observed variables included in the imputation model. We included all the predictor variables in the MICE process, along with the diagnosis of symptomatic KOA in CHARLS2011 and in CHARLS2015, as this information provides a stronger correlation structure among covariates used as predictors in the imputation model. Continuous variables (including systolic blood pressure, diastolic blood pressure, triacylglycerol, HDL cholesterol, and fasting blood glucose) were imputed using linear regression, and binary and multiple categorical variables (including duration and frequency of physical activity, history of hip fracture, smoking behavior, self-rated health status, CESD-10 items, and ADL/IADLs items) were imputed using logit regression.

Descriptive statistics (means and standard deviations for continuous data, and counts and percentages for categorical data) were used to report key variables. Univariable and multivariable logistic regression analyses were used to establish a model for predicting the risk of KOA. All candidate variables were first evaluated via an unconditional univariable logistic regression analysis, and we then selected variables according to clinical value combined with statistical significance to conduct multivariable logistic regression analysis. In the multivariable logistic regression analysis, stepwise selection was combined with the Akaike information criterion (AIC) to determine the final model structure. The coefficients, odds ratios (ORs), and 95% CIs were estimated via 1000-replication bootstrapping to obtain stable and unbiased parameters [44]. We combined the estimates using Rubin’s rules [45].

#### Internal validation

The multivariable models were internally validated using a bootstrap procedure (sampling with replacement for 1000 iterations) to assess bias-corrected estimates of predictive ability.

#### Model performance

We assessed the predictive performance of the final model using calibration and discrimination measures. Discrimination refers to the ability to distinguish patients experiencing an event from those not experiencing the event and was quantified based on the area under the receiver operating characteristic curve (AUC) in this study. Calibration refers to how closely the predicted risk corresponds with the observed risk and was assessed visually using calibration plots.

#### Clinical scoring tool

We developed a points-based risk-scoring tool based on the final model for easy clinical use—a widely utilized method of clinical scoring [23]. This clinical risk prediction tool can be used to identify individuals who are at high risk of developing KOA during the following 4 years. Continuous factors were categorized based on the results of meta-analyses and clinical practice guidelines. Scores for categorical variables were determined by multiplying the β coefficients (log odds) in the multivariable logistic regression model by ten and rounding off decimal place. The total score was calculated by summing the scores of all variables. Sensitivity, specificity, and the AUC were calculated at different cut-off values, and the maximal Youden index was used to identify the optimal cut-off point [46]. The Youden index was calculated as follows: sensitivity + specificity − 1.

The present study was conducted in accordance with the Transparent Reporting of a Multivariable Prediction Model for Individual Prognosis or Diagnosis (TRIPOD) guidelines for model development and reporting. All analyses were performed using STATA version 15.1 (STATA Corporation, College Station, TX) and R version 3.6.3 (R Foundation for Statistical Computing, Vienna, Austria). All statistical tests were two-sided and *P* values of < 0.05 were considered statistically significant.

### Ethics statement

Given that the present study is a secondary analysis of publicly available CHARLS data, the Medical Ethics Board Committee of Peking University granted the study an exemption from review.

## Results

In CHARLS2011, physical activity measures were available for 3684 participants, while blood samples were available for 11,847 participants. Complete KOA data were available in CHARLS2011 and CHARLS2015 for 9204 of these participants. Seven participants were excluded because they declined to undergo body measurements assessments, rendering over 50% of participant’s variables (including measurements of weight, height, waist circumference, assessments of depressive symptoms, physical activity, ADLs/IADLs, or the blood biomarkers) inaccessible. Among them, one patient was diagnosed with KOA in 2011, and one developed KOA in 2015. Among the remaining 9197 participants, an additional 1004 were excluded because they were diagnosed with KOA at baseline (CHARLS2011). Thus, data from a total of 8193 patients were included when developing the model. Among the 8193 included participants, 815 developed symptomatic KOA in the following 4 years. The overall 4-year cumulative incidence of symptomatic KOA was 9.95%, with 7.62% and 13.77% in males and females respectively.

The mean age was 58.82 years (standard deviation (SD) ± 9.01 years), and 4251 patients were female (51.89%). At baseline, 23.31% participants had difficulty with ADLs/IADLs, while 17.08% were diagnosed with metabolic syndrome, and 44.66% reported one or two chronic comorbidities. A history of hip fracture was reported by 252 (3.08%) participants. Other baseline characteristics are summarized in Table 1, along with the number of missing values for each variable.

**Table 1:** Baseline characteristics and outcomes of the study cohort summarized by their count and fraction (*N* (%)) for categorical or the mean and standard deviation for continuous variables, respectively

Characteristics	Number of imputed data points	Total (*N* = 8193)	No symptomatic KOA (*N* = 7378)	Symptomatic KOA (*N* = 815)	*P*
Age, mean (SD) years	0	58.82 (9.01)	58.73 (9.06)	59.60 (8.45)	< 0.01
Sex	0				< 0.01
Male		3942 (48.11)	3664 (49.66)	278 (34.11)	
Female		4251 (51.89)	3714 (50.34)	537 (65.89)	
BMI, mean (SD) kg/m^2^	0	23.46 (3.49)	23.44 (3.47)	23.47 (3.48)	0.805
Waist circumference, mean (SD) cm	0	85.31 (9.58)	85.34 (9.54)	85.12 (9.56)	0.521
Residence area	0				< 0.01
Rural, %		5406 (65.98)	4816 (65.28)	590 (72.39)	
Urban, %		2787 (34.02)	2562 (34.72)	225 (27.61)	
Smoke behavior	40				< 0.01
No smoking, %		4945 (60.36)	4374 (59.28)	571 (70.06)	
Ex-smoking, %		687 (8.39)	624 (8.46)	63 (7.73)	
Current smoking, %		2561 (31.26)	2380 (32.26)	181 (22.21)	
ADL/IADL difficulty	16				< 0.01
No, %		6283 (76.69)	5787 (78.44)	496 (60.86)	
Yes, %		1910 (23.31)	1591 (21.56)	319 (39.14)	
MS	1312				0.708
No, %		6794 (82.92)	6122 (82.98)	672 (82.45)	
Yes, %		1399 (17.08)	1256 (17.02)	143 (17.55)	
Hip fracture	72				< 0.01
No, %		7941 (96.92)	7167 (97.14)	774 (94.97)	
Yes, %		252 (3.08)	211 (2.86)	41 (5.03)	
Depression	1427				< 0.01
No, %		6132 (74.84)	5702 (77.28)	430 (52.76)	
Mild, %		1896 (23.14)	1561 (21.16)	335 (41.1)	
Moderate-to-severe, %		165 (2.01)	115 (1.56)	50 (6.13)	
Comorbidities	0				< 0.01
None, %		3778 (46.11)	3498 (47.41)	280 (34.36)	
1~2, %		3659 (44.66)	3253 (44.09)	406 (49.82)	
≥ 3, %		756 (9.23)	627 (8.5)	129 (15.83)	
Health status	3				< 0.01
Very good, %		289 (3.53)	278 (3.77)	8 (0.98)	
Good, %		1075 (13.12)	1037 (14.06)	38 (4.66)	
Fair, %		2752 (33.59)	2538 (34.4)	214 (26.26)	
Poor, %		2957 (36.09)	2607 (35.33)	350 (42.94)	
Very poor, %		1120 (13.67)	915 (12.4)	205 (25.15)	
Physical Activity Score					
VPA score	4743				0.507
No PA, %		4942 (60.32)	4462 (60.48)	480 (58.9)	
Low level, %		590 (7.20)	524 (7.1)	66 (8.1)	
Middle-to-high level, %		2661 (32.48)	2392 (32.42)	269 (33.01)	
MPA score	4750				< 0.01
No PA, %		3192 (38.96)	2914 (39.5)	278 (34.11)	
Low level, %		891 (10.88)	798 (10.82)	93 (11.41)	
Middle-to-high level, %		4110 (50.16)	3666 (49.69)	444 (54.48)	
LPA score	4751				0.842
No PA, %		1586 (19.36)	1431 (19.4)	155 (19.02)	
Low level, %		1389 (16.95)	1245 (16.87)	144 (17.67)	
Middle-to-high level, %		5218 (63.69)	4702 (63.73)	516 (63.31)	

*ADL*, activities of daily living, *BMI* body mass index, *IADL* instrumental activities of daily living, *LPA* light physical activity, *MPA* moderate physical activity, *MS* metabolic syndrome, *PA* physical activity, *SD* standard deviation, *VPA* vigorous physical activity

### Univariable and multivariable analysis

Table 2 shows the results of the univariable and multivariable analysis based on the imputed datasets. In the univariable analysis, age was identified as a risk factor for KOA, with the biggest difference occurring between the 60–64 and 65–69 age groups. Female sex, rural residence, history of hip fracture, ADL/IADL difficulty, severe depressive symptoms, more chronic comorbidities, poor health status, and higher levels of moderate physical activity (MPA) were significantly associated with an increased risk of developing KOA (all *P* values ≤ 0.01), while smoking was significantly associated with a decreased risk of developing KOA (*P* ≤ 0.01). Although high BMI/waist circumference and metabolic syndrome were also positively associated with the incidence of KOA, these associations were not significant (all *P* values > 0.05). Considering the important effects of metabolism and vigorous physical activity on the incident of KOA, we included metabolic syndrome and level of vigorous physical activity (VPA) in the multivariable logistic model, although the significance was not significant. As the clinical values of BMI and waist circumference are comparable, we selected waist circumference into the multivariable logistic regression given the relatively smaller *P* values.

**Table 2:** Results of logistic regression models for incident of KOA generated from 8193 participants in CHARLS 2011–2015

Predictors	Total *N*	KOA case n (%)	Univariable OR (95% CI)	Multivariable OR (95% CI)
Age (years)				
45–49	1616	132 (8.17)	Reference	Reference
50–54	1241	112 (9.02)	1.12 (0.86–1.45)	1.06 (0.81–1.39)
55–59	1773	161 (9.08)	1.12 (0.88–1.43)	1.00 (0.78–1.29)
60–64	1477	183 (12.39)	1.59 (1.26–2.01)**	1.38 (1.08–1.77)**
65–69	989	121 (12.23)	1.57 (1.21–2.03)**	1.31 (1.00–1.72)**
≥ 70	1097	106 (9.66)	1.20 (0.92–1.57)	0.95 (0.71–1.26)
Sex				
Male	3942	278 (7.05)	Reference	Reference
Female	4251	537 (12.63)	1.91 (1.64–2.22)**	1.51 (1.22–1.87)***
BMI level				
Normal weight	318	35 (11.01)	Reference	–
Under weight	5198	503 (9.68)	1.15 (0.80–1.66)	–
Overweight	2201	225 (10.22)	1.06 (0.90–1.25)	–
Obese	476	52 (10.92)	1.14 (0.85–1.55)	–
Waist circumference, cm in male/female				
< 85/80	3313	307 (9.27)	Reference	Reference
85~90/80~85	1414	144 (10.18)	1.11 (0.90–1.37)	1.07 (0.86–1.33)
90~95/85~90	1348	141 (10.46)	1.14 (0.93–1.41)	1.06 (0.85–1.33)
> 95/90	2118	223 (10.53)	1.15 (0.96–1.38)	1.06 (0.86–1.32)
Residence area				
Urban	2787	225 (8.07)	Reference	Reference
Rural	5406	590 (10.91)	1.39 (1.19–1.64)**	1.24 (1.05–1.47)*
Smoke behavior				
No smoking	4945	571 (11.55)	Reference	Reference
Ex-smoking	687	63 (9.17)	0.77 (0.59–1.02)	0.98 (0.71–1.35)
Current smoking	2561	181 (7.07)	0.58 (0.49–0.69)**	0.85 (0.68–1.08)
ADL/IADL difficulty				
No	6283	496 (7.89)	Reference	Reference
Yes	1910	319 (16.7)	2.34 (2.01–2.72)**	1.49 (1.26–1.76)***
MS				
No	6794	672 (9.89)	Reference	Reference
Yee	1399	143 (10.22)	1.13 (0.68–1.89)	1.03 (0.86–1.23)
Hip fracture				
No	7941	774 (9.75)	Reference	Reference
Yes	252	41 (16.27)	1.80 (1.28–2.53)**	1.53 (1.07–2.20)*
Depression				
No	6132	430 (7.01)	Reference	Reference
Mild	1896	335 (17.67)	2.85 (2.44–3.32)**	2.04 (1.73–2.41)***
Moderate-to-severe	165	50 (30.3)	5.77 (4.08–8.15)**	3.34 (2.32–4.82)***
Comorbidities				
None	3778	280 (7.41)	Reference	Reference
1~2	3659	406 (11.1)	1.56 (1.33–1.83)**	1.25 (1.05–1.48)*
≥ 3	756	129 (17.06)	2.57 (2.05–3.22)**	1.54 (1.19–1.98)**
Health status				
Very good	286	8 (2.8)	Reference	Reference
Good	1075	38 (3.53)	1.27 (0.59–2.769)	1.13 (0.52–2.46)
Fair	2752	214 (7.78)	2.93 (1.43–6.00)**	2.21 (1.07–4.54)*
Poor	2957	350 (11.84)	4.67 (2.29–9.50)**	2.99 (1.46–6.13)**
Very poor	1120	205 (18.3)	7.77 (3.79–15.98)**	3.67 (1.76–7.63)***
Physical Activity Score				
VPA score				
No PA	4942	480 (9.71)	Reference	Reference
Low level	590	66 (11.19)	1.17 (0.89–1.54)	1.15 (0.86–1.53)
Middle-to-high level	2661	269 (10.11)	1.05 (0.89–1.22)	1.03 (0.87–1.22)
MPA score				
No PA	3192	278 (8.71)	Reference	Reference
Low level	891	93 (10.44)	1.22 (0.95–1.56)	1.35 (1.02–1.73)*
Middle-to-high level	4110	444 (10.8)	1.27 (1.08–1.49)**	1.31 (1.10–1.56)**
LPA score				
No PA	1586	155 (9.77)	Reference	–
Low level	1389	144 (10.37)	1.07 (0.84–1.36)	–
Middle-to-high level,	5218	516 (9.89)	1.01 (0.84–1.22)	–

*P ≤ 0.05, **P ≤ 0.01, ***P < 0.001; *ADL* activities of daily living, *BMI* body mass index, *95% CI* 95% confidence interval, *IADL* instrumental activities of daily living, *LPA* light physical activity, *MPA* moderate physical activity, *MS* metabolic syndrome, *OR* odds ratio, *PA* physical activity, *VPA* vigorous physical activity

The final prediction model included ten variables: age, sex, waist circumference, residential area, ADLs/IADLs difficulty, history of hip fracture, depressive symptoms, number of chronic comorbidities, health status, and level of MPA.

### Model performance

**Fig. 1:**
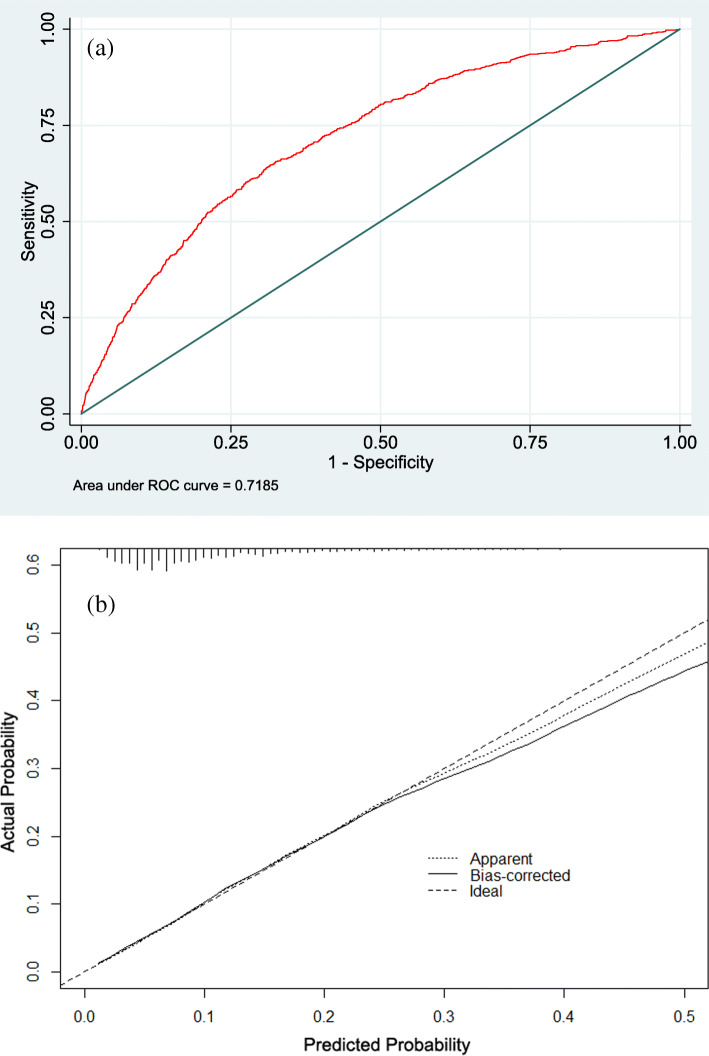
The discrimination and calibration curves of final risk model. **a** ROC curve analysis for predicting symptomatic KOA when using age, sex, waist circumference, residential area, ADL/IADL difficulty, history of hip fracture, depressive symptoms, number of chronic comorbidities, health status, and level of MPA. The AUC was 0.719 (95% CI 0.700–0.737), and optimism-corrected AUC was 0.712 after bootstrap validation. **b** The calibration curve. Area under the receiver characteristic curve, AUC; receiver operating characteristic curve, ROC

The discrimination and calibration curves for the model are shown as Fig. 1a and b, respectively. The final prediction model achieved acceptable discrimination, AUC = 0.719 (95% CI, 0.700–0.737), with optimism = 0.007 and bias-corrected AUC = 0.712 after bootstrap validation. The apparent observed line was quite close to the ideal line, while the bias-corrected line was slightly further from the ideal line than the observed line after the bootstrap procedure.

### Clinical score model

We developed a simple clinical score model based on the ten variables included in the final multivariable model (Table 3). Total scores in this model range from 0 (lowest risk) to 51 (greatest risk). This clinical score model may aid in identifying patients at the greatest risk for developing KOA within the next 4 years. The AUC of the risk score model was 0.713 (95% CI, 0.695–0.731), and the optimal cut-off, where patients with a score ≥ 20.5 were most likely to develop KOA in 4 years, was obtained from the maximal Youden index. At the optimal cut-off, the sensitivity and specificity were 63.3% and 66.0%, respectively. Referring to the previous score model [22], the incident probability of KOA within 4 years was calculated by dividing the total risk score by 51 and multiplying by 100%.

**Table 3:** Risk score model of KOA incident prediction

Predictors	Risk score	Predictors	Risk score
Age (years)		Depression	
45–49	0	No	0
50–59	1	Mild	7
60–69	3	Moderate-to-severe	12
≥ 70	0	Comorbidities	
Sex		None	0
Male	0	1 ~ 2	2
Female	5	≥3	4
Waist circumference, cm in male/female		Health Status	
< 85/80	0	Very good	0
≥ 85/80	1	Good	1
Residence Area		Fair	8
Urban	0	Poor	11
Rural	2	Very poor	13
ADLs/IADLs difficulty		MPA score	
No	0	No PA	0
Yes	4	Low level	3
Hip fracture		Middle-to-high level	3
No	0		
Yes	4		

Total score of 0 indicates lowest risk and score of 51 indicates greatest risk

A cut-off of 20.5 was identified, which meant patients with a 20.5 or more total risk scores were at the greatest risk to develop symptomatic KOA in 4 years

The incident probability of KOA within 4 years was calculated by the total risk score divided by 51 and multiplied by 100%

*MPA* moderate physical activity, *AD* activities of daily living, *IADL* instrumental activities of daily living

## Discussion

We developed and internally validated a model for predicting the 4-year risk of symptomatic KOA among the Chinese population, based on data from the CHARLS cohort. An easy-to-use clinical score model was developed to identify individuals’ risk of developing KOA. The model included ten convenient and accessible variables, including age, sex, and waist circumference, which are most commonly included in previous KOA prediction models. Besides we also included the other controversial or new predictors of KOA, which were first time tested in risk model of KOA. To our knowledge, this is the first model for predicting KOA risk in the Chinese population, and our results suggest that this model can be used to aid in the prevention of KOA.

Older age was identified as a risk factor for KOA in our study; the most significant increase in risk was observed in the 60–69 years group. The cumulative incidence of symptomatic KOA gradually increased from 45 years of age, increasing rapidly after 55 years of age, peaking at approximately 65 years of age [47]. After 70 years of age, increases in the cumulative incidence of KOA were no longer significant [47]. Our findings, along with previous, highlight the need to prevent the incident of KOA in individuals between 45 and 70 years of age.

Obesity creates an abnormal loading environment for weight-bearing joints and may contribute to the pathogenesis of KOA [48]. Alternatively, the increased risk of KOA may be caused by the positive energy balance and metaflammation associated with obesity [49]. Although BMI has been illustrated as an important predictor of KOA [50], Wallace et al. (2019) [48] reported that increased abdomen size is associated with a greater risk of radiographic KOA than high BMI. Further studies are required to determine whether BMI, waist circumference, or metabolic syndrome comprehensively influences KOA risk due to mechaflammation and metaflammation. In this study, we analyzed the effects of BMI, waist circumference, and metabolic syndrome on KOA incident in the Chinese population. None of these three factors were a significant predictor of KOA incident; BMI had relatively low significance compared with waist circumference.

We analyzed the likelihood that the damage by BMI on joint tissues and pain symptoms would not reach a significant effect in the short term. Only the 12-year Nottingham KOA model investigated BMI related to symptomatic KOA [16], and the 9-year Rotterdam model [21] and 4-year Chingford model [22] were predictive for radiographic KOA. BMI was also not included in final 4-year Chingford model. Zheng & Chen [50] synthesized that BMI was a significant factor for incident KOA, but the diagnosis of KOA was radiographic KOA or severe KOA or replacement of KOA in 13 of 14 included studies. Among the eight studies with follow-up duration less than 10 years, none focused on symptomatic KOA. Another possible reason was that abnormal waist circumference was much prevalent than abnormal BMI in the Chinese population because body feature is prone to be small in the Asian race compared with the European or American population [51], thus waist circumference was much significant with incident KOA than BMI in this study. The results imply that the sensitivity of index of obesity might vary with race when evaluating the risk of KOA. Additional studies focusing on risk model of KOA are required to verify the significance of BMI with the incident of symptomatic KOA in Chinese population and other ethnic populations.

Another conventional risk factor for KOA was physical activity. In the present study, no significant association was observed between VPA/light physical activity (LPA) and the incident of KOA; however, MPA positively predicted the incident of KOA. The reported associations between physical activity and the incident of KOA were inconsistent, resulting from a variation in assessment methods, activity categories, or populations. Felson et al. [28] reported walking and other recreational activities did not increase the risk of OA in older adults. Results from the Chingford cohort demonstrated that physical activities related to work and sports increase the risk of osteophytes, while walking decreases the risk of osteophytes in middle-aged women [52]; however, all effects were not statistically significant. Findings from the Framingham Heart Study [53] indicate that performing over 2 h/day would increase risk symptomatic KOA (OR, 5.3; 95% CI, 1.2–24) and the association was also significant for radiographic KOA (OR, 1.3 per hour; 95% CI, 1.1–1.6), while the effects of MPA and LPA were insignificant. Given the discrepancy between studies, additional studies should aim to verify the influence of different types of physical activity on the risk of KOA. Such studies should seek to determine the most appropriate type, duration, frequency, and intensity of physical activity for preventing KOA in different populations.

In our model, health-related variables are addressed and our findings provide evidence that these variables contribute essential values to the prediction of symptomatic KOA. Depressive symptoms, comorbidities, and history of hip fracture are psychologically and physiologically objective factors related to KOA. Self-rated health and difficulty with ADL/IADLs were mainly subjective, which was reflected in the patient’s knowledge and ability to cope with disease.

Patients with KOA are prone to be comorbid with depression and other chronic comorbidities, and chronic diseases often exhibit interactions with comorbidities in complex ways [54]. Hence, previous studies have assumed that there may be a potential effect of depression and chronic comorbidities on incident of KOA. Seavey et al. [34] indicated that depressive symptoms represented a risk factor for arthritis incident (OR, 1.72; 95% CI, 1.27–2.35). Jinks et al. [55] also reported that depression was a significant predictor of knee pain (OR, 1.4; 95% CI, 1.1–1.8), where pain is the dominant physical symptom among patients with symptomatic KOA. Our study is the first model involving depression as predictor in a prediction model of symptomatic KOA. Patients with mild or moderate-to-severe depression were two or three times more likely to develop KOA than those without depression. Although a bidirectional causal association has rarely been illustrated either between arthritis and depression or between any other chronic disease and depression, targeted strategies for addressing depressive symptoms may therefore aid in reducing the incident of KOA. We also assessed relationships for 12 main types of comorbidities with incident of KOA. KOA and comorbidities may accelerate the progression of one another [24]. Results showed that patients with comorbidities had a significantly increased risk of developing KOA within 4 years. This addressed the effect of comorbidities in developing KOA in the Chinese population; this might have some value for developing prediction model of KOA in other ethnic groups.

Related studies [56, 57] have demonstrated that rheumatoid arthritis increases the risk of hip fracture due to bone loss induced by chronic inflammation, use of glucocorticoids, and physical inactivity. However, rare studies indicated the association between hip fracture and KOA incident. Given that the knee and hip joints are the two most important weight-bearing joints, we sought to determine whether a history of hip fracture increases the risk of developing KOA. Our findings indicated that a history of hip fracture was associated with a 53% increase in the risk of KOA. Identifying the potential mechanisms underlying this association should be helpful for development of risk model in further studies.

Patient-reported outcome (PRO) has been emphasized in multiple studies because PROs may capture important disease-related information prior to the onset of clinical signs or pathophysiological changes [58]. Silverwood et al. [24] noted that poor self-rated health status was a potential risk factor for KOA in an earlier study, although the association was insignificant. Our model showed that the likelihood of developing KOA increased as health status worsened and impairments in ADLs/IADLs. Self-rated health status and assessments of difficulty with ADLs/IADLs could be significant predictors for incident of symptomatic KOA. Self-ratings of health status comprehensively reflect one’s physical and psychological function, as well as one’s knowledge and ability to cope with diseases and self-efficiency. Most of the existing potential risk factors were pooled from epidemiological analyses or clinicians’ experience. Our findings highlight the need to consider the patient’s perspective, as this may aid in furthering our understanding of KOA while reducing the incidence of the disease. Symptomatic KOA progressively decreases self-care ability, causing knee pain or stiffness. Our results implied that impairments in ADLs/IADLs prior to KOA onset may represent a predictive signal for KOA. Hence, preventive interventions may be useful in reducing the incident of KOA in those who have difficulty with ADL/IADL. Improving ADLs/IADLs might become a new interventional target to prevent KOA.

Preventing KOA or other chronic disease in rural area is the biggest challenge in China because of large population [36]. Our model included resident area as one predictor for KOA aiming to improve the prevention of KOA in rural population. Factors related to the high prevalence in rural areas may be multiple, including limited access to knowledge regarding the prevention of KOA and other chronic diseases, a lack of economic resources for timely treatment of chronic diseases, poor ability to manage one’s health, and earlier impairments in physical function due to strenuous farm work. We hope that our results could promote policies and resources directed toward preventing KOA in Chinese rural areas in future.

We developed an easy-to-use clinical score model to identify risk of symptomatic KOA within 4 years and to identify individuals at high risk. This model involved ten commonly available variables. This simple model involved ten commonly available variables, the assessment of variables, and the calculation of risk score, which are both easily understood and handled in practice. Clinicians or the patients themselves could use this tool to assess the risk of KOA in 4-year term. While the score model showed a good performance in assessing risk of KOA (AUC = 0.713; 95% CI 0.695–0.731), the ability to identify individuals at high risk was moderate using a − 20.5 cut-off. Hence, this model should be improved and adjusted when applied in other populations. Adding other clinical biomarkers would provide further insight.

Limitations of the study include the incomplete data, though which was handed using the imputation method and a bootstrap strategy, may have biased our findings, especially the physical activity with a high percentage missing. Second, while new variables included in the study were significantly associated with the incident of KOA, further studies are required to elucidate the mechanisms underlying these associations. Lastly, our model was internally validated; therefore, external validation in other Chinese populations and different ethnic groups remains necessary.

## Conclusion

In the present study, we developed the first model for predicting the 4-year risk of developing symptomatic KOA in China, using longitudinal cohort. Our simple score model may aid in the early identification of individuals at the greatest risk of developing KOA within 4 years in clinical practice or community setting. Such early identification may allow for improved patient education and modification of certain risk factors, which may in turn decrease rates of KOA incident.

## Supplementary Information

**Additional file 1: Supplementary Box 1.** Potential predictors and methods of measurement.

**Additional file 2: Supplementary Figure 1.** Flowchart of study participants.

**Additional file 3: Supplementary Table 1.** Baseline characteristic in excluded participants and included participants.

**Additional file 4: Supplementary Table 2.** Multiple Testing Results of Logistic Regression Models.

## Data Availability

The datasets used and analyzed during the current study are available from the corresponding author on reasonable request.

## Abbreviations

ADLs: Activities of daily living
AIC: Akaike information criterion
AUC: Area under the receiver operating characteristic curve
BMI: Body mass index
CESD: Center for Epidemiologic Studies Depression Scale
CHARLS: China Health and Retirement Longitudinal Study
CI: Confidence intervals
IADLs: Instrumental activities of daily living
KOA: Knee osteoarthritis
LPA: Light physical activity
MPA: Moderate physical activity
MS: Metabolic syndrome
OR: Odds ratio
PA: Physical activity
PRO: Patient-reported outcome
SD: Standard deviation
TRIPOD: Transparent Reporting of a Multivariable Prediction Model for Individual Prognosis or Diagnosis
VPA: Vigorous physical activity
