# Supplementary material for: Development of a model for predicting the 4-year risk of symptomatic knee osteoarthritis in China: a longitudinal cohort study
Source: Arthritis Res Ther. 2021 Feb 26;23:65. doi: 10.1186/s13075-021-02447-5 (PMC7908741; doi:10.1186/s13075-021-02447-5)
Supplement: Supplementary file 1 — Additional file 1: Supplementary Box 1. Potential predictors and methods of measurement. [file 13075_2021_2447_MOESM1_ESM.docx]

Supplementary Box 1. Potential predictors and methods of measurement

| - Sex (male vs. female) - Age at baseline (CHARLS2011), which was classified as follows: 45–49 years, 50–54 years, 55–59 years, 60–64 years, 65–69 years, ≥70 years - Smoking status (non-smoker, ex-smoker, current smoker) - Body mass index (BMI), which was calculated as measured weight (kg) divided by height squared (m^2^). BMI was then categorized as underweight (<18.5 kg/m^2^), normal (18.5–24.9 kg/m^2^), overweight (25.0–29.9 kg/m^2^), or obese (≥30.0 kg/m^2^) based on the ranges established by the World Health Organization (WHO). - Waist circumference was measured in centimeters at the height of the umbilicus. According to the diagnosed criteria of central obesity recommended by the Department of Disease Control at the Ministry of Health (Chen, Lu, & Department of Disease Control Ministry of Health, 2004), waist circumference was categorized into four groups: <85/80 cm, <90/85 cm, <95/90 cm, and ≥95/90 cm in men and women, respectively. And the first group referred to the normal group. - The residential area was categorized as either “rural” or “urban” and confirmed based on household and community IDs recorded in CHARLS. - History of hip fracture was assessed based on responses to the following question: “The history of hip fracture is assessed via the question “Have you ever fractured your hip?” Participants responding in the affirmative were considered to have a history of hip fracture. - Depressive symptoms were assessed using the CESD-10, the Cronbach’s α of which reached 0.815. Responses to items on the CESD-10 are rated along a four-point scale that ranges from 0 (rarely or none of the time/<1 day) to 3 (most or all of the time/5–7 days). Total CESD-10 scores range from 0 to 30, with higher scores indicating more severe depressive symptoms. Based on the study conducted by Cheng & Chan (2005)，the cutoff of depressive symptoms was 12 or more scores with CESD-10. In this study, scores of ≤11, 12–23, and ≥24 points were considered to reflect no, mild, or moderate-to-severe depressive symptoms. - Physical activity was classified into three levels according to intensity in CHARLS: (a) vigorous physical activity (VPA): activities that require high-intensity physical effort and make one breathe much harder than normal (e.g. heavy lifting, digging, aerobics, fast bicycling, cycling with a heavy load, etc.); (b) moderate physical activity (MPA): activities that can result in breathing somewhat harder than normal (e.g. carrying light loads, bicycling at a regular pace, mopping the floor); (c) light physical activity (LPA): walking, including walking at work, traveling from place to place, and walking for recreation. Participants were asked whether they engaged in VPA, MPA, or LPA for at least 10 continuous minutes in a typical week. Participants responding in the affirmative were asked to report how many days they engaged in each type of activity and how much time spent engaged in each (<30 min, <2 h, <4 h, ≥4 h). Each time/duration was encoded as 1–4. The frequency of VPA/MPA/LPA ranged from 0–7 days/week and was classified into the following three groups: 1–2 days, 3–5 days, 6–7 days, which were coded as 1–3, respectively. The physical activity score was then calculated by multiplying the duration by the frequency (MacInnis et al., 2004): VPA/MPA/LPA score = frequency of VPA/MPA/LPA × duration of VPA/MPA/LPA. Final physical activity scores were classified as follows: no PA (0 points), low PA (1–4 points), moderate-high PA (5–12 points). - Comorbidities were assessed based on self-reports of physician-diagnosed chronic diseases including hypertension, dyslipidemia, diabetes or high blood sugar (i.e. impaired glucose tolerance or elevated fasting blood glucose), cancer or malignant tumor (excluding minor skin cancers), chronic lung diseases (excluding tumors or cancer), liver disease, heart disease (including heart attack, coronary heart disease, angina, congestive heart failure, or other heart problems), stroke, kidney disease (except for tumor or cancer), stomach or other digestive diseases (except for tumor or cancer), memory-related diseases, and asthma. We classified the number of comorbidities into three categories: none, 1~2, ≥3. - Metabolic syndrome (MS) was defined in accordance with Chinese Diabetes Society (CDS) criteria (Metabolic Syndrome Research Group of Chinese Medical Association, 2004), based on the presence of any three or more of the following four components: (a) overweight/obesity, with BMI ≥25 kg/m^2^; (b) dyslipidemia: hypertriglyceridemia ≥1.7 mmol/L (150 mg/dL) or low high-density lipoprotein cholesterol (HDL-C) <0.9 mmol/L (35 mg/dL) in men and <1.0 mmol/L (39 mg/dL) in women; (c) high blood pressure: systolic blood pressure (SBP) ≥140 mmHg, diastolic blood pressure (DBP) ≥90 mmHg, or use of antihypertensive medication; or (d) hyperglycemia: fasting plasma glucose (FPG) ≥6.1 mmol/L (110 mg/dL) and/or 2-h plasma glucose ≥7.8 mmol/L (140 mg/dL) and/or current treatment for diabetes. - Self-rated health status was assessed based on responses to one of the following two questions, which were asked randomly: (a) “Would you say your health is excellent, very good, good, fair, or poor?”; (b) “Would you say your health is very good, good, fair, poor, or very poor?” Responses were assigned scores ranging from 1 to 5, with higher values indicative of poorer health status. - ADL/IADL difficulty was assessed using the Katz ADL (Katz, Ford, Moskowitz, Jackson, & Jaffe, 1963) and the Lawton IADL (Lawton, Lawton, Brody, & Brody, 1969). ADLs refer to daily self-care tasks including taking a bath, eating, getting in and out of bed, dressing, using the toilet and maintaining continence of urine and feces. IADLs refer to abilities such as doing housework, cooking, taking medicine, shopping and taking care of finances, which are required for living independently in the community. Responses were provided using the following scale: 1: No, I do not have any difficulty; 2: I have difficulty but can still do it; 3: Yes, I have difficulty and need help; 4: I cannot do it. Participants who provided responses 2 to 4 to any item were considered to have difficulty with ADLs or IADLs, being consistent with the definition that has been used in previous studies (Connolly, Garvey, & McKee, 2017; Feng et al., 2013). |
| --- |

**Reference**

Chen, C., Lu, F. C., & Department of Disease Control Ministry of Health, P. R. C. (2004). The guidelines for prevention and control of overweight and obesity in Chinese adults. *Biomedical and environmental sciences : BES, 17*, 1-36.

Connolly, D., Garvey, J., & McKee, G. (2017). Factors associated with ADL/IADL disability in community dwelling older adults in the Irish longitudinal study on ageing (TILDA). *Disability and rehabilitation, 39*(8), 809-816. doi:10.3109/09638288.2016.1161848

Feng, Q., Zhen, Z., Gu, D., Wu, B. E. I., Duncan, P. W., & Purser, J. L. (2013). Trends in ADL and IADL Disability in CommunityDwelling Older Adults in Shanghai, China, 1998―2008. *The journals of gerontology. Series B, Psychological sciences and social sciences, 68*(3), 476-485.

Katz, S., Ford, A. B., Moskowitz, R. W., Jackson, B. A., & Jaffe, M. W. (1963). STUDIES OF ILLNESS IN THE AGED. THE INDEX OF ADL: A STANDARDIZED MEASURE OF BIOLOGICAL AND PSYCHOSOCIAL FUNCTION. *JAMA : the journal of the American Medical Association, 185*, 914-919.

Lawton, M. P., Lawton, M. P., Brody, E. M., & Brody, E. M. (1969). Assessment of older people: Self-maintaining and instrumental activities of daily living. *Gerontologist, 9*(3), 179-186. doi:10.1093/geront/9.3_Part_1.179

MacInnis, R. J., English, D. R., Hopper, J. L., Haydon, A. M., Gertig, D. M., & Giles, G. G. (2004). Body Size and Composition and Colon Cancer Risk in Men. *Cancer Epidemiology Biomarkers & Prevention, 13*(4), 553-559.

Cheng, S.-T., & Chan, A. C. M. (2005). The Center for Epidemiologic Studies Depression Scale in older Chinese: thresholds for long and short forms. *International journal of geriatric psychiatry, 20*(5), 465-470. doi:10.1002/gps.1314

Metabolic Syndrome Research Group of Chinese Medical Association. (2004). Suggestions on Metabolic Syndrome from Chinese Diabetes Society. *Chinese Journal of Diabetes Mellitus*, 12(03): p. 5-10.
