# Supplementary figures and images for: Development of a model for predicting the 4-year risk of symptomatic knee osteoarthritis in China: a longitudinal cohort study
Source: Arthritis Res Ther. 2021 Feb 26;23:65. doi: 10.1186/s13075-021-02447-5 (PMC7908741; doi:10.1186/s13075-021-02447-5)

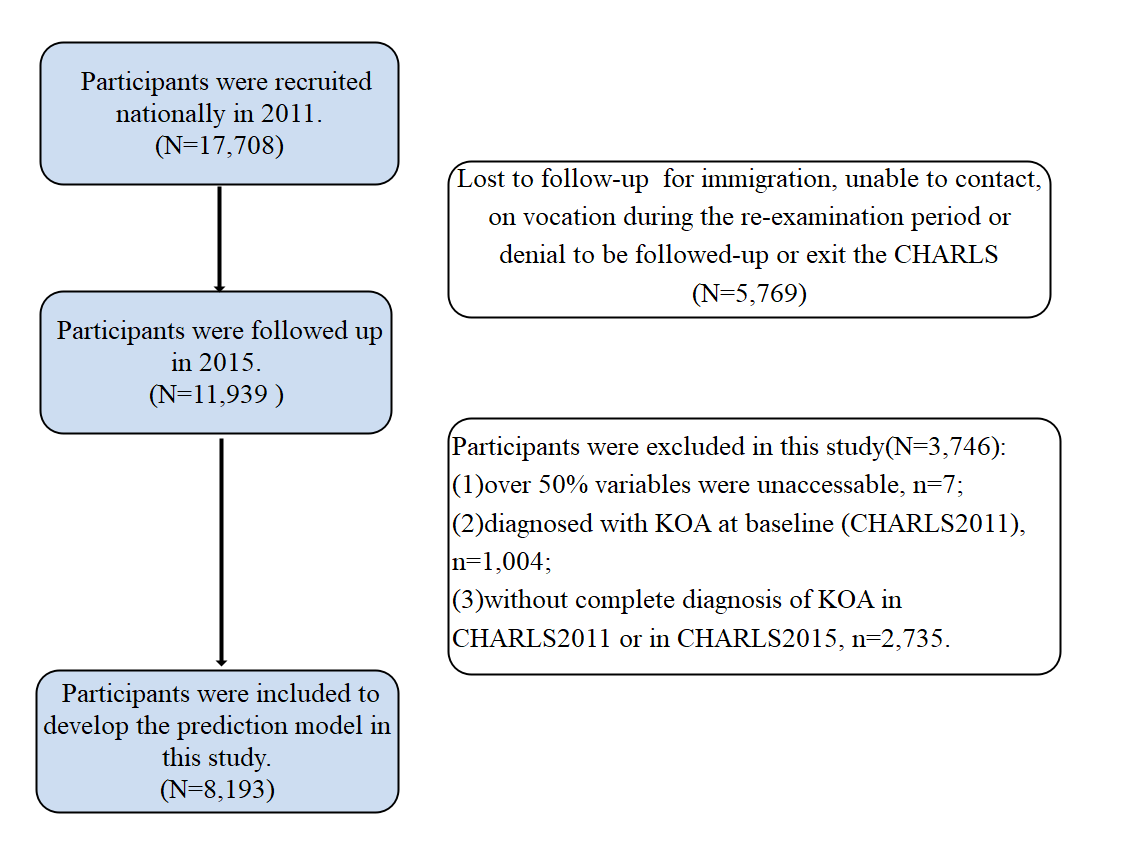


Supplementary Figure 1. Flowchart of study participants.

Supplement: Supplementary file 2 — Additional file 2: Supplementary Figure 1. Flowchart of study participants. [file 13075_2021_2447_MOESM2_ESM.docx]
