# Supplementary material for: Development of a model for predicting the 4-year risk of symptomatic knee osteoarthritis in China: a longitudinal cohort study
Source: Arthritis Res Ther. 2021 Feb 26;23:65. doi: 10.1186/s13075-021-02447-5 (PMC7908741; doi:10.1186/s13075-021-02447-5)
Supplement: Supplementary file 3 — Additional file 3: Supplementary Table 1. Baseline characteristic in excluded participants and included participants. [file 13075_2021_2447_MOESM3_ESM.docx]

Supplementary Table 1. Baseline characteristic in excluded participants and included participants (N=17,708)

| Variables in baseline | Participants Excluded  （N1=9515） | | Participants Included  （N2=8193） | | *p*-value |
| --- | --- | --- | --- | --- | --- |
|  | N | % | N | % |  |
| Gender |  |  |  |  | 0.450 |
| Male | 4,536 | 47.67 | 3,942 | 48.11 |  |
| Female | 4,979 | 52.33 | 4,251 | 51.89 |  |
| Age-Mean (SD), year | 59.25 | 11.05 | 58.82 | 9.01 | **0.040** |
| BMI- Mean (SD), kg/m^2^ | 23.33 | 3.47 | 23.46 | 3.49 | 0.115 |
| Waist circumference-Mean (SD), cm | 85.21 | 16.5 | 85.12 | 9.56 | 0.503 |
| Residence Area |  |  |  |  | **<0.001** |
| Rural | 5,131 | 53.93 | 5,406 | 65.98 |  |
| Urban | 4,384 | 46.07 | 2,787 | 34.02 |  |
| Smoke Behavior |  |  |  |  | **0.020** |
| No smoking | 2,898 | 60.06 | 4,923 | 60.38 |  |
| Ex-smoking | 473 | 9.80 | 685 | 8.40 |  |
| Current smoking | 1,454 | 30.13 | 2,545 | 31.22 |  |
| ADL/IADL Difficulty |  |  |  |  |  |
| No | 6,683 | 70.13 | 6,272 | 76.70 | **<0.001** |
| Yes | 2,847 | 29.87 | 1,905 | 23.30 |  |
| MS |  |  |  |  |  |
| No | 2,876 | 84.81 | 5,918 | 86.00 | 0.107 |
| Yes | 515 | 15.19 | 963 | 14.00 |  |
| Hip Fracture |  |  |  |  |  |
| No | 7,970 | 98.14 | 8,010 | 98.63 | **<0.001** |
| Yes | 151 | 1.86 | 111 | 1.37 |  |
| Number of comorbidities-Mean (SD) | 1.11 | 1.27 | 0.91 | 1.27 | **<0.001** |
| Depression scores | 10.38 | 5.03 | 9.64 | 5.03 | **<0.001** |
| Health Status |  |  |  |  |  |
| Very good | 304 | 3.23 | 286 | 3.49 | **<0.001** |
| Good | 1,111 | 11.82 | 1,075 | 13.13 |  |
| Fair | 2,820 | 29.99 | 2,752 | 33.60 |  |
| Poor | 3,359 | 35.72 | 2,957 | 36.11 |  |
| Very poor | 1,809 | 19.24 | 1,120 | 12.68 |  |
| Physical Activity Level |  |  |  |  |  |
| VPA Score |  |  |  |  | **<0.001** |
| No PA | 2381 | 69.28 | 2176 | 63.07 |  |
| Low level | 199 | 5.79 | 212 | 6.14 |  |
| Middle-to-high level | 857 | 24.93 | 1062 | 30.78 |  |
| MPA Score |  |  |  |  | **<0.001** |
| No PA | 1,649 | 48.06 | 1,381 | 40.11 |  |
| Low level | 349 | 10.17 | 365 | 10.60 |  |
| Middle-to-high level | 1,433 | 41.77 | 1,697 | 49.29 |  |
| LPA Score |  |  |  |  | **<0.001** |
| No PA | 738 | 21.66 | 666 | 19.35 |  |
| Low level | 642 | 18.84 | 570 | 16.56 |  |
| Middle-to-high level | 2,027 | 59.50 | 2,206 | 64.09 |  |

Continuous variables (age, BMI, waist circumference, number of comorbidities, and depression scores) were reported as mean with standard deviations (SD), and other categorical variables were expressed as number of patients (%). Independent t-test was used to assess differences of continuous variables between groups. The differences in the composition of categorical variables were identified using Pearson’s chi-squared tests and Fisher’s exact test between groups.

ADL = Activities of daily living; BMI = Body mass index; IADL = Instrumental activities of daily living; LPA = Light physical activity; MPA = Moderate physical activity; MS = Metabolic syndrome; PA = Physical activity; SD = Standard deviation; VPA = Vigorous physical activity.
