# Supplementary material for: Development of a model for predicting the 4-year risk of symptomatic knee osteoarthritis in China: a longitudinal cohort study
Source: Arthritis Res Ther. 2021 Feb 26;23:65. doi: 10.1186/s13075-021-02447-5 (PMC7908741; doi:10.1186/s13075-021-02447-5)
Supplement: Supplementary file 4 — Additional file 4: Supplementary Table 2. Multiple Testing Results of Logistic Regression Models. [file 13075_2021_2447_MOESM4_ESM.docx]

Supplementary Table 2. Multiple Testing Results of Logistic Regression Models for Incident of KOA Generated from 8193 Participants in CHARLS 2011–2015

| Predictors | Total N | KOA case  n (%) | Univariable OR  (99% CI) | Multivariable OR (99% CI) | p-value |
| --- | --- | --- | --- | --- | --- |
| Age (years) |  |  |  |  |  |
| 45-49 | 1,616 | 132(8.17) | Reference | Reference | — |
| 50-54 | 1,241 | 112(9.02) | 1.12(0.79–1.58) | 1.06(0.74–1.51) | 0.679 |
| 55-59 | 1,773 | 161(9.08) | 1.12(0.82–1.54) | 1.00(0.78–1.29) | 0.983 |
| 60-64 | 1,477 | 183(12.39) | 1.59(1.27–2.17) *** | 1.38(1.00–1.91) | 0.010 |
| 65-69 | 989 | 121(12.23) | 1.57(1.11–2.20) *** | 1.31(0.95–1.87) | 0.050 |
| ≥70 | 1,097 | 106(9.66) | 1.20(0.85–1.71) | 0.95(0.65–1.38) | 0.709 |
| Sex |  |  |  |  |  |
| Male | 3,942 | 278(7.05) | Reference | Reference | — |
| Female | 4,251 | 537(12.63) | 1.91(1.56–2.33) *** | 1.51(1.14–2.01) | <0.001 |
| BMI level |  |  |  |  |  |
| Normal weight | 318 | 35(11.01) | Reference | — | — |
| Under weight | 5,198 | 503(9.68) | 1.15(0.77–1.70) | — | — |
| Overweight | 2,201 | 225(10.22) | 1.06(0.85–1.32) | — | — |
| Obese | 476 | 52(10.92) | 1.14(0.72–1.86) | — | — |
| Waist circumference, cm  In male/female |  |  |  |  |  |
| <85/80 | 3,313 | 307(9.27) | Reference | Reference | — |
| 85~90/80~85 | 1,414 | 144(10.18) | 1.11(0.84–1.46) | 1.07(0.80–1.42) | 0.552 |
| 90~95/85~90 | 1,348 | 141(10.46) | 1.14(0.87–1.51) | 1.06(0.29–1.43) | 0.615 |
| >95/90 | 2,118 | 223(10.53) | 1.15(0.91–1.46) | 1.06(0.80–1.40) | 0.590 |
| Residence Area |  |  |  |  |  |
| Urban | 2,787 | 225(8.07) | Reference | Reference | — |
| Rural | 5,406 | 590(10.91) | 1.39(1.13–1.72) *** | 1.24(0.98–1.53) | 0.018 |
| Smoke Behavior |  |  |  |  |  |
| No smoking | 4,945 | 571(11.55) | Reference | Reference | — |
| Ex-smoking | 687 | 63(9.17) | 0.77(0.54–1.11) | 0.98(0.65–1.50) | 0.916 |
| Current smoking | 2,561 | 181(7.07) | 0.58(0.12–0.15) *** | 0.85(0.63–1.16) | 0.185 |
| ADL/IADL Difficulty |  |  |  |  |  |
| No | 6,283 | 496(7.89) | Reference | Reference | — |
| Yes | 1,910 | 319(16.7) | 2.34(1.92–2.85) *** | 1.49(1.19–1.85) | <0.001 |
| MS |  |  |  |  |  |
| No | 6,794 | 672(9.89) | Reference | Reference | — |
| Yee | 1,399 | 143(10.22) | 1.13(0.89–1.36) | 1.03(0.76–1.34) | 0.231 |
| Hip Fracture |  |  |  |  |  |
| No | 7,941 | 774(9.75) | Reference | Reference | — |
| Yes | 252 | 41(16.27) | 1.80(1.15–2.82) *** | 1.53(0.95–2.46) | 0.02 |
| Depression |  |  |  |  |  |
| No | 6,132 | 430(7.01) | Reference | Reference | — |
| Mild | 1,896 | 335(17.67) | 2.85(2.33–3.48) *** | 2.04 (1.65–2.53) | <0.001 |
| Moderate-to-severe | 165 | 50(30.3) | 5.77(3.66–9.09) *** | 3.34(2.06–5.40) | <0.001 |
| Comorbidities |  |  |  |  |  |
| None | 3,778 | 280(7.41) | Reference | Reference | — |
| 1~2 | 3,659 | 406(11.1) | 1.56(1.26–1.92) *** | 1.25(1.00–1.55) | 0.011 |
| ≥3 | 756 | 129(17.06) | 2.57(1.91–3.46) *** | 1.54(1.10–2.16) | 0.001 |
| Health Status |  |  |  |  |  |
| Very good | 286 | 8(2.8) | Reference | Reference | — |
| Good | 1,075 | 38(3.53) | 1.27(0.46–3.52) | 1.13(0.41–3.15) | 0.752 |
| Fair | 2,752 | 214(7.78) | 2.93(1.14–7.51) ** | 2.21(0.86–5.70) | 0.031 |
| Poor | 2,957 | 350(11.84) | 4.67(1.83–11.89) *** | 2.99(1.16–7.70) | 0.003 |
| Very poor | 1,120 | 205(18.3) | 7.77(3.03–20.03) *** | 3.67(1.40–9.63) | <0.001 |
| Physical Activity Score |  |  |  |  |  |
| VPA score |  |  |  |  |  |
| No PA | 4,942 | 480(9.71) | Reference | Reference | — |
| Low level | 590 | 66(11.19) | 1.17(0.82–1.68) | 1.15(0.79-1.68) | 0.350 |
| Middle-to-high level | 2,661 | 269(10.11) | 1.05(0.85–1.29) | 1.03(0.83-1.29) | 0.708 |
| MPA score |  |  |  |  |  |
| No PA | 3,192 | 278(8.71) | Reference | Reference | — |
| Low level | 891 | 93(10.44) | 1.22(0.88–1.69) | 1.35(0.945–1.88) | 0.032 |
| Middle-to-high level | 4,110 | 444(10.8) | 1.27(1.03–1.56) ** | 1.31 (1.05–1.63) | 0.002 |
| LPA score |  |  |  |  |  |
| No PA | 1,586 | 155(9.77) | Reference | — | — |
| Low level | 1,389 | 144(10.37) | 1.07(0.78–1.46) | — | — |
| Middle-to-high level | 5,218 | 516(9.89) | 1.01(0.79–1.30) | — | — |

*P≤0.05, **P≤0.01, ***P<0.001; ADL= Activities of daily living; BMI = Body mass index; 95% CI=95% Confidence interval; IADL= Instrumental activities of daily living; LPA = Light physical activity; MPA = Moderate physical activity; MS= Metabolic syndrome; OR= Odds ratio; PA = Physical activity; VPA = Vigorous physical activity.
